# Specialization Does Not Predict Individual Efficiency in an Ant

**DOI:** 10.1371/journal.pbio.0060285

**Published:** 2008-11-18

**Authors:** Anna Dornhaus

**Affiliations:** 1 Ecology and Evolutionary Biology, University of Arizona, Tucson, Arizona, United States of America; 2 School of Biological Sciences, University of Bristol, Bristol, United Kingdom; University of London, Queen Mary College, United Kingdom. Professor Chittka was the graduate advisor of Dr. Dornhaus

## Abstract

The ecological success of social insects is often attributed to an increase in efficiency achieved through division of labor between workers in a colony. Much research has therefore focused on the mechanism by which a division of labor is implemented, i.e., on how tasks are allocated to workers. However, the important assumption that specialists are indeed more efficient at their work than generalist individuals—the “Jack-of-all-trades is master of none” hypothesis—has rarely been tested. Here, I quantify worker efficiency, measured as work completed per time, in four different tasks in the ant Temnothorax albipennis: honey and protein foraging, collection of nest-building material, and brood transports in a colony emigration. I show that individual efficiency is not predicted by how specialized workers were on the respective task. Worker efficiency is also not consistently predicted by that worker's overall activity or delay to begin the task. Even when only the worker's rank relative to nestmates in the same colony was used, specialization did not predict efficiency in three out of the four tasks, and more specialized workers actually performed worse than others in the fourth task (collection of sand grains). I also show that the above relationships, as well as median individual efficiency, do not change with colony size. My results demonstrate that in an ant species without morphologically differentiated worker castes, workers may nevertheless differ in their ability to perform different tasks. Surprisingly, this variation is not utilized by the colony—worker allocation to tasks is unrelated to their ability to perform them. What, then, are the adaptive benefits of behavioral specialization, and why do workers choose tasks without regard for whether they can perform them well? We are still far from an understanding of the adaptive benefits of division of labor in social insects.

## Author Summary

Social insects, including ants, bees, and termites, may make up 75% of the world's insect biomass. This success is often attributed to their complex colony organization. Each individual is thought to specialize in a particular task and thus become an “expert” for this task. Researchers have long assumed that the ecological success of social insects derives from division of labor, just as the increase in productivity achieved in human societies; however, this assumption has not been thoroughly tested. Here, I have measured task performance of specialized and unspecialized ants. In the ant species studied here, it turns out that specialists are no better at their jobs than generalists, and sometimes even perform worse. In addition, most of the work in the colony is not performed by the most efficient workers. So the old adage “The Jack of all trades is a master of none” does not seem to apply to these ants, suggesting that we may have to revise our understanding of the benefits of colony organization

## Abstract 3

The assumption that the success of social insects rests on the increased efficiency of dividing tasks within the colony is challenged by evidence that specialists are not always better at their jobs.

## Introduction

Social insects are enormously successful ecologically. Ants, social bees, social wasps, and termites may make up 75% of the world's insect biomass, they play a major role in soil turnover and nutrient cycling, and they often surpass vertebrates in their biomass in a habitat [1]. Division of labor is often cited as the primary reason for the ecological success of social insects, particularly ants [1–5]. Division of labor implies that individuals within a colony specialize on particular tasks, such as brood care, foraging, nest building, or defense, and conversely that each task is performed by a particular subset of the workers [6–12]. If division of labor caused ecological success in social insects, it must have conferred benefits to colonies. What exactly are these adaptive benefits of specialization? According to the famous economist Adam Smith [13], specialization in human industry had three benefits: (1) increased individual efficiency through learning, (2) reduction of switching costs, and (3) the invention of machines. The first of these may be called the “Jack-of-all-trades is master of none” hypothesis: specialists are individually more efficient at performing their task than generalists. Although this hypothesis underlies many discussions of division of labor [2,6,14–18], it has rarely been tested, as pointed out by many authors [8,9,11,17,19–27]. Here, I address this issue by measuring individual efficiency of more than 1,100 workers of the ant species Temnothorax albipennis in several tasks. This allows me to test whether more specialized individuals are also more efficient.

Most previous research on division of labor in insects concentrates on the mechanisms of task allocation (e.g., [6,11,27–32]) instead of its consequences for individual or colony-level performance. Remarkably few studies have investigated the efficiency of individuals and how it relates to which tasks they perform [9,15,22,23,25,33–36]. In principle, there are two ways in which individuals who are specialists may achieve higher efficiency in performing “their” task: they may learn to perform a task better with frequent experience; or colonies may produce different specialists that are evolutionarily adapted to particular tasks. Worker polymorphism may be such an evolutionary adaptation: in ants with morphological castes, we know that “majors” (morphologically specialized ants) tend to be better at some tasks than the generalist “minors,” for example, they may be good at cutting leaves or walking fast to transport them [35,37]; they may also be good at other transport, defense, or food storage: [25,38–41], but are bad at performing brood care [22]. Polymorphism among workers, however, is rare, only occurring in less than 15% of ant genera [21,42]. Worker polymorphism also does not occur in bees or wasps. In bumble bees, workers exhibit size polymorphism, albeit not the variation in shape (allometry) characteristic of polymorphic ants. Workers may also differ genetically, leading to variation in behavior [43]. Although this may be unlikely to produce evolutionarily stable colony-level benefits [44], it is clear that worker differences in task preferences [30,31,45], as well as variation in quality of task performance [31,46,47], may be linked to genetic variation, and such variation may therefore play a role in specialization.

Individual efficiency can also increase through learning, without morphological adaptations. For example, bumble bees and honey bees need to invest time in learning to handle particular flower types efficiently [48,49]. Since learning incurs various types of cost (production and maintenance of neural tissue, energy costs of actually using it, and costs in errors made and time invested [50–55]), it may be beneficial to minimize the number of skills that an individual has to learn. This could lead to increased individual efficiency in specialists. Indeed, many bee foragers specialize on particular flower types, possibly to minimize costs of learning handling procedures [48,56,57] (although see [52]).

So, is the specialist worker in a colony the “master” of one task, while the generalist is a “master of none”? The Jack-of-all-trades is a master of none hypothesis would predict that more specialized workers perform a task with higher efficiency than generalists, whether this is a result of learning or adaptation. This hypothesis is what I test here for the ant Temnothorax albipennis. A set of tasks that are relevant in different contexts (foraging, emigrations, and nest building) will be used. Specifically, I will test which of the following specific hypotheses best predicts individual efficiency: (1) More specialized workers perform a task more efficiently. (2) Workers that are more active overall (in different tasks) perform tasks more efficiently. (3) Workers that engage in tasks with a short delay (who may have low response thresholds) perform tasks more efficiently than those who delay longer.

To understand the role that individual efficiency plays in colony division of labor, I will also test the following two hypotheses: (1) At the colony level, most labor is contributed by highly efficient workers. (2) At the colony level, most labor is contributed by specialized workers.

## Results

**Figure 1:**
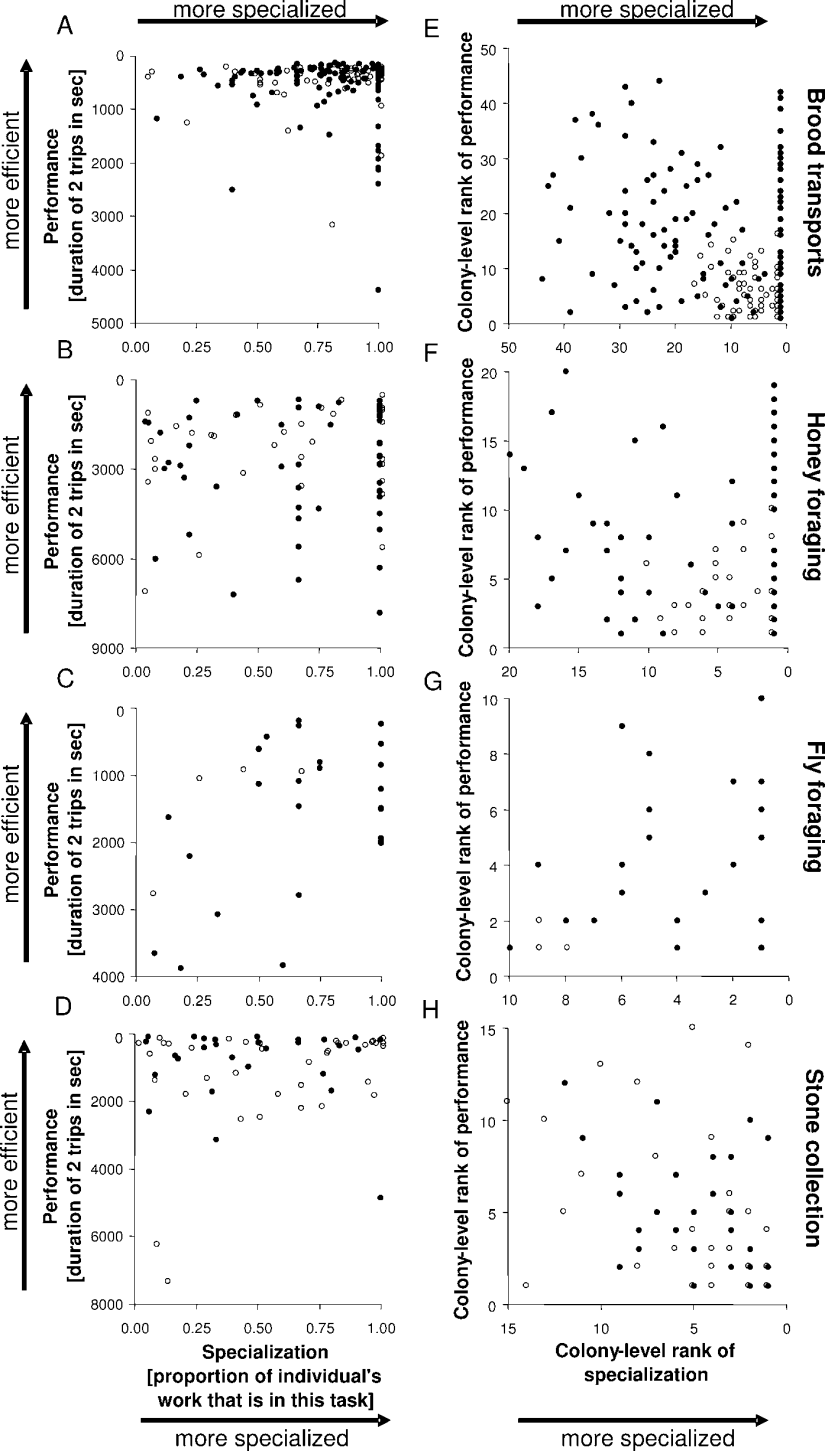
The Relationship between Efficiency of Task Performance in and Specialization on a Task (A–D) show raw data, (E–H) show ranks within colonies, of task efficiency (*y*-axis) and specialization (*x*-axis; here defined as percentage of total task performances by this ant that are in the focal task). Each data point is an individual ant, filled circles are ants in large colonies, and open circles are in small colonies (open circles are offset slightly). A high rank on the *y*-axis means a high transport rate (short trip duration); a high rank on the *x*-axis reflects a low degree of specialization. Ants with a rank of 1 for specialization were only seen performing that task (100% of their task performances are the focal task). If more specialized individuals were more efficient at performing tasks, there would be negative correlations both in the raw data plots (shorter time to perform two trips with higher specialization) and in the rank plots (although in both cases, the plots are arranged so that a specialized, well-performing worker would fall in the upper-right corner). Regression for all workers of colony-specific ranked specialization on ranked trip duration: brood transporting—large colonies: *p* = 0.12, *R* ^2^ = 0.01, *df* = 115; small colonies: *p* = 0.17, *R* ^2^ = 0.01, *df* = 64; honey foraging—large colonies: *p* = 0.92, *R* ^2^ < 0.001, *df* = 55; small colonies: *p* = 0.74, *R* ^2^ < 0.001, *df* = 31; fly foraging—large colonies: *p* = 0.23, *R* ^2^ = 0.02, *df* = 24; small colonies: *n* = 4 ants, no test performed; stone collection—large colonies: *p* = 0.03, *R* ^2^ = 0.13, *df* = 27; small colonies: *p* = 0.01, *R* ^2^ = 0.15, *df* = 36.

A total of 1,142 ants from 11 colonies of Temnothorax albipennis were marked individually and filmed performing four tasks: carrying brood items in a colony emigration, foraging for honey solution, foraging for protein (dead *Drosophila* flies), and collecting sand grains (hereafter, “stones”—they are about a third the size of a worker ant) as nest-building material. The four tasks studied have in common that it is possible to measure the amount of work performed per time by individual workers, without manipulation of colony composition, which may upset normal task allocation patterns. Of the colonies, four were large relative to the average colony size [58] in this species (147–233 workers) and seven were small (27–100 workers). These colonies were also used in other studies, in which colony size effects on individual workload in emigrations and the difference between “elite workers” and “specialized workers” were investigated ([12] and A. Dornhaus, J.-A. Holley, and N. R. Franks, unpublished data). In the study presented here, I focus on the quality of performance of individuals. For each individual ant, it was recorded how often it performed each task, how long it delayed before starting to perform the task (“delay,” see Materials and Methods), and how long it took the ant to perform two task units. A task unit (hereafter: “trip”) is defined as leaving the nest and returning to it while delivering either a brood item to a new nest site, a load of honey solution to a nestmate, a piece of fly (i.e., proteinaceous food) to the nest, or a stone to the wall being built. Thus, the duration per trip reflects how much work a worker accomplished per time, and therefore can be used as a performance measure. I calculated the average duration per trip for the first two trips of each ant that performed a task (hereafter called “performance”). For each ant and each task, a measure of specialization was also calculated: an ant was considered more specialized the more it concentrated its work effort in a single task. I used the proportion of total task performances (trips) that were in task X as a measure of specialization on task X. Thus, if all performances were in a single task, the worker's specialization for that task was 100%; if a task was never performed, specialization was 0%; and if all four tasks were performed equally frequently, specialization was 25%. Using these measures and ranking workers within each colony according to their performance, I find no correlation between specialization and performance for three tasks: brood transporting, honey foraging, and fly foraging (Figure 1). This means that in each colony, the workers that were the most specialized in a task were not necessarily the best performers. In the fourth task, collection of stones, I did find a significant impact of specialization on colony-level rank of performance (Figure 1; regression for large colonies: *p* = 0.03, *R* ^2^ = 0.13, *df* = 27; small colonies: *p* = 0.01, *R* ^2^ = 0.15, *df* = 36). However, this relationship was not in the direction predicted: workers with a high rank on specialization (mostly performing stone collection) had a high rank on duration of two trips, which means they were less efficient (took longer to perform the same amount of work). Note also that these results remain unchanged if workers who performed a task only once are excluded (large colonies: brood transports, *p* = 0.062; small colonies: brood transports, *p* = 0.38; honey foraging, *p* = 0.64; stone collection, *p* = 0.041; in no case were more than two workers excluded; if *p*-value is not given here, all workers performed the task at least twice or not at all).

Instead of specialization, it may be that the overall number of trips across all tasks predicts a worker's performance. To test this, I performed a stepwise regression of performance on three factors: overall activity level (total number of trips in all tasks performed by that worker), task-specific delay (time from start of experiment to first task performance), and specialization. None of these factors consistently predicted performance (Table 1). Only in one case (performance of brood transports in small colonies) was specialization a significant factor, although even here, activity, i.e., amount of work performed overall rather than in a specific task, was more predictive of performance, and only a small amount of the variation in performance was explained by either of these two factors.

**Table 1:**
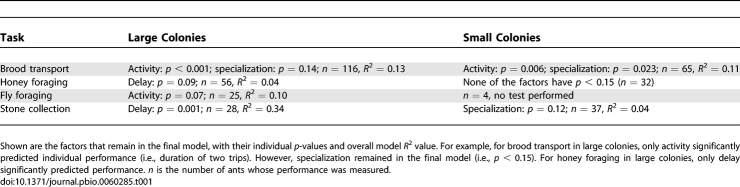
Stepwise Regression of Performance on Activity, Delay, and Specialization

Another puzzling result is that I do not find that the contribution a worker makes to overall colony workload is predicted by worker efficiency (Table 2). This means that in a given colony, most of the work is not necessarily performed by those who are best at it; it is, however, often performed by those most specialized in a task (Table 2). This agrees with a previous result that work in Temnothorax albipennis is generally performed by specialists, not generalists (A. Dornhaus, J.-A. Holley, and N. R. Franks, unpublished data).

**Table 2:**
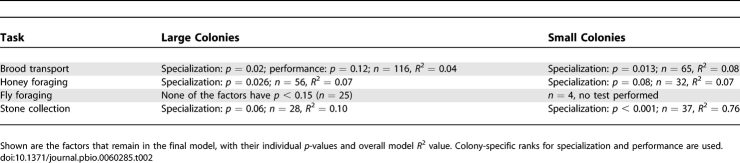
Stepwise Regression of Colony-Level Work Contribution on Performance and Specialization

**Figure 2:**
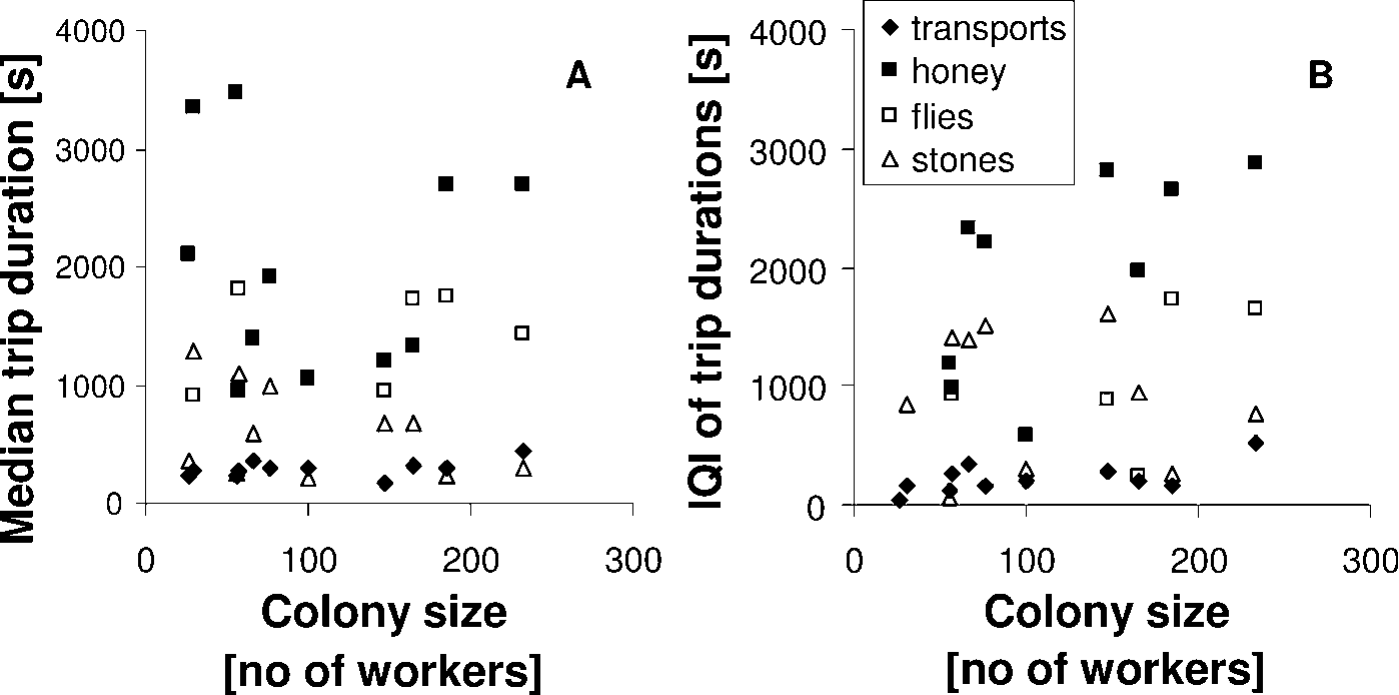
No Effect of Colony Size on Individual Efficiency or Variation in Efficiency among Workers Each colony is represented by one data point. For each ant, the average trip duration across the first two trips is calculated; for each colony, the median (A) and the interquartile range (B) across all ants in the colony is shown. Different tasks (brood transports in emigrations, honey foraging, fly foraging, and stone collection for nest building) are shown in different symbols. Both transport duration and variance are highest for honey foraging, and lowest for brood transports in emigrations.

Colonies did not differ significantly in how well their workers performed, except in the task of brood transports (Kruskal-Wallis Test, large colonies, transports: *p* = 0.024, *df* = 3, *n* = 115 workers; honey foraging: *p* = 0.47, *n* = 56, fly foraging: *p* = 0.38, *n* = 25; stone collection: *p* = 0.20, *n* = 28; small colonies, transports: *p* = 0.36, *df* = 6, *n* = 64 workers; honey foraging: *p* = 0.016, *n* = 32, stone collection: *p* = 0.26, *n* = 37). Median individual performance for colonies does not seem to depend on colony size (Figure 2A; brood transports: *p* = 0.15, *R* ^2^ = 0.13; honey foraging: *p* = 0.89, *R* ^2^ < 0.001; fly foraging: *p* = 0.58, *R* ^2^ < 0.001; stone collection: *p* = 0.20, *R* ^2^ = 0.08); variation, measured as the interquartile interval, among individuals also seems mostly constant even in different-sized colonies, although it was significantly higher in larger colonies for brood transports (Figure 2B; brood transports: *p* = 0.048, *R* ^2^ = 0.30; honey foraging: *p* = 0.06, *R* ^2^ = 0.33; fly foraging: *p* = 0.44, *R* ^2^ < 0.001; stone collection: *p* = 0.69, *R* ^2^ < 0.001).

**Figure 3:**
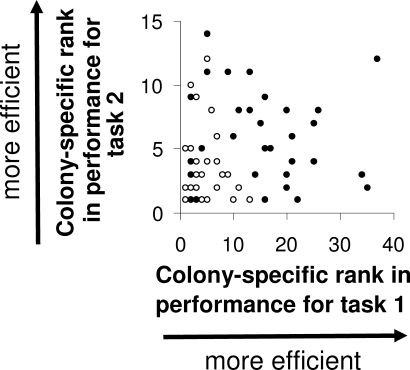
No Consistency in Performance across Tasks All individuals observed in more than any two tasks are shown. Workers from large colonies: filled circles; from small colonies: open circles. Each individual's rank among nestmates in two tasks is graphed. If individual workers were either high- or low-performing across all tasks, we would see a positive correlation here. That there is no significant correlation implies that performance in one task does not predict performance in another task.

Some individuals were only seen performing one task (35% of all workers in large, 32% in small colonies), and many were never seen performing any of the four tasks investigated here (52% of all workers in large, 57% in small colonies; see also [12] for inclusion of more tasks). Thus, the experimental conditions did not simply induce all workers to work at maximum level, which would have potentially obscured a normal relationship between specialization and individual performance. For workers whose performance was measured in at least two different tasks, their colony-specific rank in performance in one task did not correlate with that in the other task (Figure 3; Regression *p* = 0.44, *R* ^2^ < 0.001, *n* = 62). As stated above, for each worker in each task, two task performances (trips) were measured: duration of the first trip correlates significantly with duration of the second trip for transports, but not for the other tasks (regression on colony-specific ranks, transports: *p* < 0.001, *R* ^2^ = 0.19, *n* = 101; honey foraging: *p* = 0.07, *R* ^2^ = 0.11, *n* = 21; fly foraging: *p* = 0.83, *R* ^2^ < 0.001, *n* = 8; stone collection: *p* = 0.18, *R* ^2^ = 0.05, *n* = 19). In honey foraging and stone collection, there was a trend in the same direction (higher rank in trip 1 ∼ higher rank in trip 2), but the sample sizes were lower than for transports; it thus cannot be said with certainty whether individuals were consistent in their performance over time.

## Discussion

Among the results presented here, two are particularly surprising: that colonies are not adapted to allocate the most efficient workers to each task, and that efficiency seems unrelated to the level of behavioral specialization of individuals. Many previous studies have simply assumed that if there is specialization, it will correlate with improved performance at a task (although see [22,23,59–61]). My results indicate that at least in this species, a task is not primarily performed by individuals that are especially adapted to it (by whatever mechanism). This result implies that if social insects are collectively successful, this is not obviously for the reason that they employ specialized workers who perform better individually. It also seems that individual performance (at least in the four tasks investigated) is not predicted by overall activity or that ant's delay to engaging in a task. Delay may or may not correspond to a task response threshold, an individual- and task-specific factor that defined the probability of engaging in a task. This factor has been used in many previous models of task allocation. It will be interesting for future studies to investigate whether response thresholds do or do not predict individual efficiency in performing tasks.

The performance measure here was the average duration of two individual trips, which corresponds to the number of items brought to the nest per time. Although this is a performance measure that is often used and can be objectively quantified, it is possible that the performance of specialist ants was superior to that of generalists in some other way. Perhaps specialists carried larger loads (although this seems unlikely in the nest-building case, as the sand grains were sieved to a uniform size), or perhaps specialists were able to collect more information or watch for predators while performing tasks. However, the time used per load, as measured here, varied by more than a factor of 40 (for example, fastest brood transport was 100 s, slowest 4,363 s). Although it cannot be excluded, it seems unlikely that these differences were compensated by load size or minimization of predation risk.

It is tempting to say that the ant species studied here, Temnothorax albipennis, is unusual in its colony organization. Maybe it employs less strict division of labor than other ant species (although other measurements indicate that this is not the case: A. Dornhaus, J.-A. Holley, and N. R. Franks, unpublished data), or maybe because of their long lifespan (workers can live several years in the lab), each individual already has had the opportunity to perfect its performance in each task. Also note that, as stated above, T. albipennis does not have worker polymorphism, so any differences in specialization among workers are the result of behavioral specialization only. However, the level of specialization in most social insect species is not known, and it can be argued that *Temnothorax* is representative of the majority of ant species: it has the same small colony sizes that are typical for most ants [1,62]; it forages by preying on and scavenging other arthropods in the leaf litter, as many other ants do [1,21]; it is monomorphic (no allometry among workers) as most other ants are [21,42]; and the genus *Temnothorax* is cosmopolitan and does not consist of ecological specialists adapted to particularly restricted habitats. To test whether the present results are widely applicable throughout the social insects, it would be desirable if future research employed a wide variety of study systems. That would enable an assessment of how widespread, across species, individual behavioral specialization is, and how it relates to efficiency.

In addition, only four tasks that ants perform on a regular basis were studied here. There are a number of other relevant tasks, most notably brood care and colony defense against predators and parasites. Efficiency assays for these tasks should be developed and used to study the benefits of specialization. Although studying other tasks is important, the tasks studied here were previously thought to be the ones that are most likely to be influenced by learning and thus suitable for specialization [30,48,63]. Tasks that involve leaving the colony require skills of orientation and the specific learning of landmarks [64,65]; even in small laboratory settings, such learning can significantly affect performance [66]. Similarly, tasks that involve collecting prey or building material involve identification and handling skills that cannot be easily genetically preprogrammed, as the precise location and type of prey and building material is likely to vary with microhabitat, even within a population (e.g., [34,63,67–69]).

Indeed the results presented here do not show that learning is absent in this species. Learning, in the context of task performance, may theoretically occur at three time scales. Short-term learning may increase performance from one trip to the next on the same day, without leading to long-term individual differences. Second, performance at foraging and emigration tasks may differ primarily between completely naive individuals who have never left the nest and individuals who have left at least once, i.e., participated in at least one emigration or foraging bout (or perhaps performed the equivalent of the well-studied “orientation flights” in honey bees, e.g., [51,64]). Third, amount of experience may directly correlate with performance, such that the more experience an individual gains at a specific task over its lifetime, the better it is able to perform a task. The results in this study show that there is no correlation between quality of performance and specialization—suggesting that differential improvement through learning, as in the third type of learning listed above, does not occur. However, it is quite likely that the first two types of learning do occur. Previous studies have demonstrated learning in colony emigrations and foraging in this species [70–75]. However, in many cases the main improvements were achieved after the first performance of the task (although see [69] for honey bees), suggesting an effect of learning similar to the second type discussed above.

In summary, this study finds that there is a large amount of variation in individual quality of task performance, not explained by any of the factors studied. The mechanism creating this variation is unknown, and may be genetic, developmental, or an effect of experience. Thus, learning may well affect task performance, but either it affects all individuals equally, or workers do not preferentially perform the tasks in which they are experienced (although the latter would contradict previous studies: [71,76,77]). Either way, learning does not seem to lead to superior performance by specialists. It will be important for future research to quantify at what time scales learning occurs, and whether it increases or decreases variance among individuals in the long term.

In this study, I quantify quality of task performance for individual workers in several tasks. To show that specialization exists, it is necessary to show that an individual performs more of one task, and less of another, compared to nestmates [10,11]. It is not sufficient to measure how much an individual performs a single task: this may merely identify high-activity workers from low-activity workers. To measure the benefits of specialization (at least in terms of individual efficiency or quality of task performance), it is equally necessary to compare performance in multiple tasks; otherwise one may simply identify high-quality workers from low-quality ones, without necessarily demonstrating that specialists are better at their task and worse (or at least no better) at another. If this is not the case, then one has demonstrated merely that there is variation in quality of task performance among individuals, and possibly that high-quality individuals tend to be allocated to particular tasks, but not that there are benefits to specialization.

What, then, are the benefits of division of labor in species without polymorphic workers? As mentioned above, there are at least three potential benefits of division of labor. Individually increased efficiency was only one of them. Others were a decrease in the costs associated with switching between tasks. For example, division of labor may lead to increased spatial efficiency, as hypothesized for ants [28], or reduction of other, possibly cognitive, switching costs [57]. It is also possible that specialization simplifies the process of task allocation (i.e., minimizes neural or other costs associated with the task selection process itself), or optimizes material flow in multistep tasks, [15]. Any of these processes may create colony-level fitness benefits from division of labor, even without improvement in individual efficiency as measured here. Future studies should attempt to quantify switching costs, spatial constraints, the role of learning, and the time scales at which individuals specialize in social insect colonies. My study also highlights that findings from commonly used model species, such as honey bees or leaf-cutting ants, which have very unusual and specific ecology and morphology, cannot necessarily be extended to other species [20,21,78]. We have much yet to learn about the benefits and evolution of division of labor.

## Materials and Methods

As mentioned above, all workers in 11 colonies of the ant species Temnothorax albipennis, collected in Dorset, England, were individually marked with paints (a total of 1,142 ants were marked; details on this method, as well as colony collection and housing, can be found in [12]). The colonies were housed in artificial nests in the laboratory, made of a cardboard perimeter sandwiched between two glass slides. All colonies were filmed in three different contexts spaced at least a week apart (emigrations, wall building, and foraging). Each colony was filmed for at least 180 min in each context, starting at the time of manipulation as described below. This resulted in 166 h of digital video tape. Each context was initiated as follows: emigration—removal of the top glass slide, exposing the ants (a new, identical nest was offered in 10 cm distance); foraging—colonies were starved for 2 wk (no food, but water ad lib was offered), and then a small dish with honey solution (1:10 honey:water) and a pile of ten frozen *Drosophila* flies were placed 10 cm from the nest entrance; building—colonies were housed in a nest that had no front wall, creating a 33-mm-wide gap; on the day after the ants had moved into this nest, and a pile of colored and sieved sand grains was offered 10 cm from the nest. Under the latter conditions, ants use the sand grains to build a wall to narrow the nest entrance to approximately 1–3 mm. No food was offered in the emigration and building contexts, and no building material was offered in the emigration and foraging contexts.

Each of the three different contexts thus introduced the need to perform a particular set of tasks, creating an opportunity for each ant to take part in these tasks. By using separate contexts, ants' task choices were thus less affected by competing stimuli for different tasks, but solely by the individual's preferences for performing the task at hand. For example, if some individuals had both a high tendency to transport brood and to participate in nest building, their activity level in either of these tasks was not constrained by that in the other task. If all tasks had been offered at once, such individuals may have spent all their time transporting brood simply because it is the more urgent task. Only a separation of tasks as employed here allows the identification of specialists from highly active generalist individuals.

From the video tapes, the time that each ant picked up a brood item in the old nest, a sand grain from the pile, or left the nest in a foraging trip was extracted (start of trip). The time from the start of the experiment (e.g., removal of the nest cover in emigration experiments) to the start of the first trip for each ant was designated its task-specific delay. Then, the time that the same ant returned to the old nest, dropped the sand grain at the nest, returned and performed trophallaxis (to unload honey solution to a nestmate), or returned with a piece of dead fly to the nest was recorded (end of trip). The time difference between start and end of trip give the trip duration. The sum of all trips made in one context by one colony was called the colony-level workload (e.g., total number of brood transports made in an emigration). An individual's colony-level work contribution was measured as that individual's number of trips divided by the total colony-level workload. All measurements were double-checked by a second person to ensure accurate records of ant identity and timing of task performances.
